# Draft Genome Sequence of Lactobacillus plantarum IYO1511, Isolated from Ishizuchi-Kurocha

**DOI:** 10.1128/MRA.00143-20

**Published:** 2020-04-30

**Authors:** Ryo Niwa, Yolani Syaputri, Masanori Horie, Hitoshi Iwahashi

**Affiliations:** a Faculty of Applied Biological Sciences, Gifu University, Gifu, Japan; b The United Graduate School of Agricultural Science, Gifu University, Gifu, Japan; c Health Research Institute, National Institute of Advanced Industrial Science and Technology (AIST), Takamatsu, Kagawa, Japan; DOE Joint Genome Institute

## Abstract

Lactobacillus plantarum IYO1511, isolated from a traditional postfermented tea, is a predominant species associated with Ishizuchi-kurocha. Here, we report the whole-genome sequence of this bacterium. The draft genome comprises 3,229,083 nucleotides and 3,044 coding DNA sequences (CDSs), with an average G+C content of 44.5%.

## ANNOUNCEMENT

Ishizuchi-kurocha is a postfermented tea produced in Saijo, Ehime, Japan. The Ishizuchi-kurocha fermentation process is complex because two major groups of microorganisms are involved in the fermentation. The primary fermentation step is aerobic, in which filamentous fungi (*Aspergillus* spp.) play a pivotal role (1, 2). The secondary fermentation step is controlled predominantly by lactobacilli. Through lactate production during the secondary fermentation, the tea acquires its unique acidic flavor and fragrance. In addition, lactate decreases the pH and prolongs the storage term of the tea. Previous investigations have revealed Lactobacillus plantarum, Lactobacillus pentosus, and Lactobacillus brevis as the predominant species associated with this tea. Currently, there are no reports on the genetic characteristics of lactic acid bacteria in the postfermented tea. In this study, we sequenced the genome of one of the strains to reveal the mystery of the dynamics in the tea.

*L. plantarum* IYO1511 was isolated from Ishizuchi-kurocha tea leaves (Camellia sinensis var. *sinensis*) after anaerobic fermentation of the tea, which was produced in Saijo, Ehime, in 2017. Wet tea leaves were mixed with sterile distilled water at a concentration of 0.1 mg/ml. The suspension was serially diluted with distilled water and spread on MRS agar plates. After that, the plates were incubated at 30°C for 1 to 2 days under anaerobic conditions. The anaerobic conditions were provided by AnaeroPack-Anaero (Mitsubishi Gas Chemical Co., Inc., Tokyo, Japan). Stand-alone colonies were picked and inoculated into 10 ml of MRS broth in a test tube with a screw cap. Each isolate was cultivated at 30°C for 1 to 2 days. Stock cultures were stored at –80°C in MRS broth containing 30% glycerol.

Genomic DNA was extracted from *L. plantarum* IYO1511 after 16 h of incubation using an Extrap Soil DNA Plus kit v2 (Nippon Steel & Sumikin EcoTech Corporation). The DNA sample was submitted to the Gifu University Next Generation Sequencer (NGS) service, which employs Illumina MiSeq sequencing. Using a Nextera XT DNA library prep kit, 150-bp paired-end reads were prepared. From this library, 1,349,246 reads were sequenced. A quality check was conducted with FastQC v0.11.8 (http://www.bioinformatics.babraham.ac.uk/projects/fastqc/), and adaptor and low-quality reads were trimmed with Trimmomatic v0.33, using a threshold of Q25 (3). The quality-filtered reads were assembled *de novo* by Unicycler v0.4.7, and the assembler produced 45 contigs. The gene annotation was conducted with DFAST v1.1.15 in the Web application version with the database (DB) type set to lactic acid bacteria (4). Default parameters were used for all software, unless otherwise specified. The final assembly yielded a genome of 3,229,083 bp with a mean G+C content of 44.5%, an *N*_50_ value of 186,297 bp, and a total of 3,044 genes. Benchmarking Universal Single-Copy Ortholog (BUSCO) v4.0.2 analyses using the lactobacillales data set showed 400 complete single-copy genes and 2 complete duplicated genes, out of 402 genes (5, 6).

The genome of *L. plantarum* IYO1511 is expected to be used for further investigation of genes which may play a role in resistance against antimicrobial substances from the *Aspergillus* genus. In addition, these data contribute to a better understanding of the microbiome of traditional postfermented tea.

### Data availability.

The BioSample, DRA/SRA, and BioProject accession numbers for the sequence reported here are SAMD00206687, DRR208996, and PRJDB9324, respectively. The GenBank accession number of the deposited genome is BLLP00000000.
